# Winning the Tug-of-War Between Effector Gene Design and Pathogen Evolution in Vector Population Replacement Strategies

**DOI:** 10.3389/fgene.2019.01072

**Published:** 2019-10-30

**Authors:** John M. Marshall, Robyn R. Raban, Nikolay P. Kandul, Jyotheeswara R. Edula, Tomás M. León, Omar S. Akbari

**Affiliations:** ^1^Division of Epidemiology and Biostatistics, School of Public Health, University of California, Berkeley, CA, United States; ^2^Innovative Genomics Institute, Berkeley, CA, United States; ^3^Section of Cell and Developmental Biology, University of California, San Diego, CA, United States; ^4^Tata Institute for Genetics and Society, University of California, San Diego, CA, United States

**Keywords:** clustered regularly interspaced short palindromic repeats, clustered regularly interspaced short palindromic repeats–associated protein 9, gene drive, homing, malaria, dengue, Zika

## Abstract

While efforts to control malaria with available tools have stagnated, and arbovirus outbreaks persist around the globe, the advent of clustered regularly interspaced short palindromic repeat (CRISPR)-based gene editing has provided exciting new opportunities for genetics-based strategies to control these diseases. In one such strategy, called “population replacement”, mosquitoes, and other disease vectors are engineered with effector genes that render them unable to transmit pathogens. These effector genes can be linked to “gene drive” systems that can bias inheritance in their favor, providing novel opportunities to replace disease-susceptible vector populations with disease-refractory ones over the course of several generations. While promising for the control of vector-borne diseases on a wide scale, this sets up an evolutionary tug-of-war between the introduced effector genes and the pathogen. Here, we review the disease-refractory genes designed to date to target *Plasmodium falciparum* malaria transmitted by *Anopheles gambiae*, and arboviruses transmitted by *Aedes aegypti*, including dengue serotypes 2 and 3, chikungunya, and Zika viruses. We discuss resistance concerns for these effector genes, and genetic approaches to prevent parasite and viral escape variants. One general approach is to increase the evolutionary hurdle required for the pathogen to evolve resistance by attacking it at multiple sites in its genome and/or multiple stages of development. Another is to reduce the size of the pathogen population by other means, such as with vector control and antimalarial drugs. We discuss lessons learned from the evolution of resistance to antimalarial and antiviral drugs and implications for the management of resistance after its emergence. Finally, we discuss the target product profile for population replacement strategies for vector-borne disease control. This differs between early phase field trials and wide-scale disease control. In the latter case, the demands on effector gene efficacy are great; however, with new possibilities ushered in by CRISPR-based gene editing, and when combined with surveillance, monitoring, and rapid management of pathogen resistance, the odds are increasingly favoring effector genes in the upcoming evolutionary tug-of-war.

## Introduction

While a dramatic reduction in malaria-related deaths was seen in the early part of this century, progress has halted since 2015 (Bhatt et al., 2015; Feachem et al., 2019), and models predict that elimination is not possible in the majority of disease-endemic countries with currently available tools (Walker et al., 2016). The burden of other vector-borne diseases is on the rise, with dengue incidence and mortality increasing in much of the world (Stanaway et al., 2016), Zika recently sweeping through Latin America and the Caribbean (O’Reilly et al., 2018), and the mosquito species responsible for vectoring these diseases, *Aedes aegypti* and *Aedes albopictus*, greatly expanding their geographic range (Kraemer et al., 2015; Lee et al., 2019).

The advent of clustered regularly interspaced short palindromic repeat (CRISPR)-based gene editing (Doudna and Charpentier, 2014) has provided exciting new opportunities for genetics-based strategies to control these diseases. In particular, it has greatly accelerated the development of “gene drive” systems, capable of biasing inheritance in their favor, and thereby spreading desired genes and fitness loads into a population (Esvelt et al., 2014; Champer et al., 2016). Two distinct strategies for applying this technology to mosquitoes are: i) “population suppression,” in which the gene drive system induces a fitness load or sex bias, thereby suppressing (and potentially eliminating) mosquito populations as it spreads, and ii) “population replacement,” in which the gene drive system is used to bias inheritance in favor of linked effector genes that render them unable to transmit a pathogen. The disease-susceptible vector population is thereby replaced with a disease-refractory one over the course of several generations. While exciting progress has been made on the population suppression approach in mosquitoes (Hammond et al., 2016; Kyrou et al., 2018), we focus on the population replacement strategy in this review, as the population suppression approach targets the vector population directly rather than the vector-pathogen interaction.

We also exclude from consideration *Wolbachia* for population replacement in this review (i.e., replacing a vector population with a *Wolbachia*-transfected one), as *Wolbachia*-mediated pathogen-blocking is induced by an endosymbiotic bacterium rather than an effector gene, and the mechanism of *Wolbachia*-induced pathogen-blocking is not yet well understood. The evolutionary tug-of-war between *Wolbachia* and pathogens is certainly relevant though, and worthy of a review in its own right. *Wolbachia* functions similarly to a gene drive system in spreading through a population, and has been shown to reduce vector competence for multiple arboviruses (Frentiu et al., 2014; Aliota et al., 2016). The mechanism of pathogen-blocking likely involves multiple pathways and competition for resources (Lindsey et al., 2018; Koh et al., 2019), though early evidence is mixed about whether natural selection favors enhanced or reduced pathogen-blocking by the endosymbiont (Hoffmann et al., 2015; Ford et al., 2019).

Gene drive strategies are immensely promising for the control of vector-borne diseases due to their ability to spread beyond their release site and to function independently of human compliance, which is a barrier for many interventions (Macias and James, 2016; Raban and Akbari, 2017; Burt et al., 2018). Significant progress has been made in recent years, both in terms of the development of gene drive systems (Gantz et al., 2015; Li et al., 2019) and of effector genes to target malaria parasites (Carballar-Lejarazú and James, 2017), several dengue virus (DENV) serotypes (Franz et al., 2006; Yen et al., 2018; Buchman et al., 2019a), chikungunya (CHIKV) (Yen et al., 2018), and Zika (ZIKV) (Buchman et al., 2019b). Nevertheless, the introduction of disease-refractory genes into a vector population sets up an understudied evolutionary tug-of-war between the anti-pathogen effector and pathogen evolution. Resistance can evolve against the gene drive technologies that support the introgression of these anti-pathogen effectors into the target population. For instance, CRISPR-based homing systems are particularly susceptible to the formation of homing-resistant alleles through inaccurate DNA repair events including non-homologous end-joining (NHEJ) and microhomology-mediated end-joining (MMEJ). These imprecise DNA repair pathways could also lead to loss of the disease-refractory gene by inaccurate DNA repair or mutational loss-of-function. In this review, we focus on pathogen resistance to effector genes, as other resistance mechanisms are well documented elsewhere (Marshall et al., 2017; Noble et al., 2017; Unckless et al., 2017).

We review the disease-refractory effectors designed to date to target the malaria parasite *Plasmodium falciparum* transmitted by *Anopheles gambiae*, and arboviruses transmitted by *Ae. aegypti*, including DENV serotypes 1–4, CHIKV, and ZIKV. Studies of pathogen resistance in response to synthetic antipathogen effectors have been limited to the laboratory, and conducted only over small time scales. Consequently, there is limited direct evidence for resistance to these technologies in the pathogen population. We therefore discuss lessons learned from the evolution of resistance to anti-pathogen drugs and potential implications for effector resistance management, emphasizing resistance concerns and design considerations that may mitigate pathogen escape variants. We discuss integrated control strategies that may decrease pathogen resistance to antipathogen effectors, including reducing the pathogen population size with traditional vector control measures or wide-scale distribution of antimalarial drugs, both of which could be used prior to and/or during a release. We conclude with a discussion of the target product profile (TPP) for gene drive systems intended for vector-borne disease control, with emphasis on the time span that the effector genes should be prevalent and functional in the vector population.

### Lessons From the Evolution of Resistance to Antimalarial Drugs

Antimalarial drugs provide a well-studied example of the evolutionary response of a mosquito-borne pathogen to selective pressure. Vector-borne pathogens alternate between their mosquito and human hosts; for antimalarial drugs, the suppressive effect is applied in the human host, while for disease-refractory genes, the suppressive effect is applied in the vector. For antimalarial drugs, efficacy is determined by their dosage and bioavailability, coverage level, and drugs used in combination. The efficacy of a disease-refractory gene will be determined by its expression level, timing, stability, and prevalence in the mosquito population; these are further influenced by the integration site, other effector genes present and synthetic elements included on the construct.

Resistance to antimalarial drugs has been documented several times over the course of the last 60–70 years (**Table 1**). Chloroquine, discovered in the 1930s, was quickly adopted for widespread treatment and prevention of malaria caused by *P. falciparum*, *Plasmodium vivax*, and other species. Resistance of *P. falciparum* to the drug was first documented in nature in the 1950s, and the effectiveness of chloroquine quickly declined as resistant strains of *P. falciparum* spread and evolved. Several mechanisms of chloroquine resistance that emerged in nature have been documented in the laboratory, mostly revolving around transport of chloroquine in and out of the parasite. Notably, mutations in a *P. falciparum* chloroquine resistance transporter gene (PfCRT) have been shown to permit the parasite to efflux chloroquine at a rate 40 times that of cells lacking the mutations (Martin et al., 2009). Several other mutations of transporter genes have been shown to have a protective effect against the drug, e.g., a chloroquine transporter protein (CG2), and an ATP-binding cassette transporter gene (PfMDR1) (Haldar et al., 2018).

**Table 1:** Origins of resistance in malaria parasite, *Plasmodium falciparum*, to monotherapy drugs.

Monotherapy application dates	Drug and its mechanism	Resistance and its mechanism
**∼1930–1950**	Chloroquine interferes with the detoxification of heme by accumulating in the digestive vacuole of *Plasmodium* (Haldar et al., 2018).	∼1950. Mutations in transporter genes enabling efflux of chloroquine: chloroquine resistance transporter (PfCRT) (Martin et al., 2009) (Haldar et al., 2018); chloroquine transporter (CG2) (Haldar et al., 2018); ABC transporter (PfMDR1) (Haldar et al., 2018).
**1953**	Pyrimethamine and sulfadoxine inhibit folate pathway (Gregson and Plowe, 2005; Hyde, 2005) by blocking dihydropteroate synthase (PfDhps) and dihydrofolate reductase (PfDhfr).	2009 (Gesase et al., 2009). Mutations in and/or amplification of PfDhps and PfDhfr genes (Shah et al., 2011; Costa et al., 2017).
**∼1960**	Piperaquine interferes with the detoxification of heme by accumulating in the digestive vacuole of *Plasmodium* (Eastman and Fidock, 2009).	2010 (Duru et al., 2016). Amplification of parasite protease genes, such as plasmepsin 2 and 3 (Haldar et al., 2018).
**1972**	Artemisinin suggested to interfere with the detoxification of heme (Eastman and Fidock, 2009).	2008 (Dondorp et al., 2009). Mutations in transporter genes, such as PfK13, enabling efflux of Chloroquine; or a change in target recognized by the parasite (Ouji et al., 2018).

Antifolate drugs, such as pyrimethamine and sulfadoxine, were developed and used for chloroquine-resistant parasites and in other settings from the 1950s onwards. However, resistance quickly emerged in nature from mutations to the dihydrofolate reductase (DHFR) and dihydropteroate synthase (DHPS) genes, which allowed antifolates to act on and disrupt the folate biosynthetic pathway (Gregson and Plowe, 2005; Hyde, 2005). Pyrimethamine and sulfadoxine were combined for use together and have since been paired with other drugs. This combination is still used in sub-Saharan Africa (SSA), and other locales where resistance is not too severe, due to its availability and affordability.

Piperaquine was developed in the 1960s and used in China and India for several years as a replacement for chloroquine, which is structurally similar. Both drugs are thought to accumulate in the digestive vacuole of the parasite and to interfere with the detoxification of heme. Resistance to piperaquine quickly evolved in nature, and the drug fell out of use as a monotherapy, and was later adopted for use in combination with artemisinin (Davis et al., 2005). Resistance to this combination has recently emerged in western Cambodia, where over 40% of combination treatments fail to eliminate parasites from patients’ blood (Duru et al., 2016); however, the mechanisms by which parasites become resistant to piperaquine remain unclear (Haldar et al., 2018).

Artemisinin, a semisynthetic drug re-discovered from Chinese traditional medicine in the 1970s, has been used to treat malaria for decades. Use of the drug as a monotherapy has been discouraged since the early 2000s given evidence of emerging resistance in nature and availability of several partner drugs (e.g., piperaquine and amodiaquine) that are effective and well tolerated in patients. Resistance to artemisinin-based combination therapy drugs (ACTs) was first reported in Southeast Asia in 2008 and has subsequently spread throughout the region (Dondorp et al., 2009). Several mechanisms of resistance are thought to be involved, and have been documented in laboratory studies, including mutations in transporter genes that result in an efflux of the drug away from its site of action in the cell, or a change in target recognized by the parasite (Ouji et al., 2018).

Curiously, resistance to ACTs has only been observed to a limited extent in SSA, where the malaria burden is greatest. One possible explanation is that there are many untreated malaria infections in SSA—some symptomatic, but many asymptomatic. If drug resistance is associated with a fitness cost in *P. falciparum*, then that cost could outweigh the benefits in a scenario where many infections go untreated. Treatment with “weaker” drugs is common in Southeast Asia, providing a fitness advantage to drug resistance there and a smaller evolutionary barrier for this trait to evolve. In sum, these observations suggest that: i) the malaria parasite *P. falciparum* has evolved resistance to a range of antimalarial drugs by multiple mechanisms; ii) combination therapies may help to slow the evolution of resistance (White and Olliaro, 1996); and iii) low-dose treatments likely provide a smaller evolutionary hurdle for the emergence of resistance.

### Malaria-Refractory Genes and Resistance Concerns

**Figure 1:**
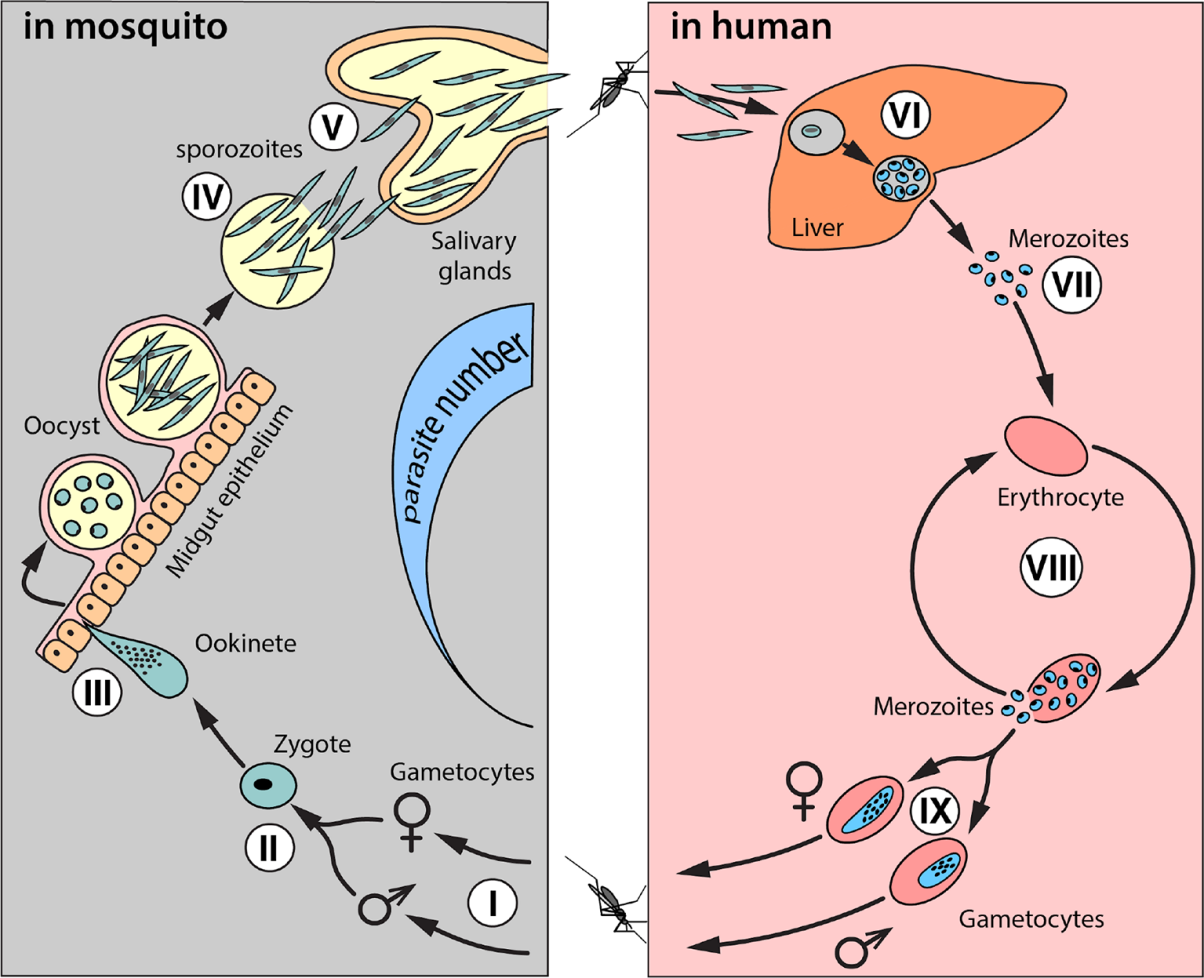
Schematic of the life cycle of *Plasmodium falciparum*. The complete life cycle takes place in two organisms: *Anopheles* mosquitoes and humans. A female mosquito ingests micro- and macro-gametocytes (I) in blood from an infectious human, which then fuse into diploid zygotes (II). Zygotes develop into ookinetes (III) that penetrate the midgut epithelium and grow into oocysts. Sporozoites (IV) multiply inside oocysts and escape into the hemolymph to migrate to the salivary glands. This process takes 10–18 days (the extrinsic incubation period). When an infected female mosquito feeds again, sporozoites are released with saliva into the human bloodstream. In humans, sporozoites migrate from the blood into liver cells where they mature, multiply, and develop into merozoites (VI). When merozoites rupture from liver cells, they infect and multiply in erythrocytes (VII). This starts cycles of synchronous erythrocyte infection/rupture typical of malaria fever (VIII). Some merozoites develop into gametocytes (IX), which can be ingested by a female mosquito, and the cycle begins again.

The malaria life cycle is complex and antimalarial effector genes could target the parasite at several stages of development in the mosquito, including: i) mosquito ingestion of gametocytes from human blood, ii) microgametocyte and macrogametocyte coalescence, iii) ookinete and oocyst formation, iv) rupture and sporozoite release into the midgut, and v) sporozoite migration to the salivary glands (**Figure 1**). Theoretically, any stage in this life cycle can be targeted; but the ookinete/oocyst life stage is a particularly attractive target, as it represents a population bottleneck for malaria parasites in the mosquito. The most effective antimalarial effector genes will likely target the parasite at multiple life stages.

Malaria-refractory genes and other putative effector strategies to target malaria parasites at various developmental stages are reviewed by Carballar-Lejarazu and James (Carballar-Lejarazú and James, 2017). These include cellular melanization mechanisms that occur in nature and inactivate the malaria parasite by encapsulating it in the midgut (Yassine et al., 2012) (Dong et al., 2011) (Dong et al., 2012), blocking receptors that the parasite recognizes and requires in order to pass through the midgut or salivary glands through genetic engineering (Ito et al., 2002), and a range of other potential targets and effectors potentiated in recent years through the advent of CRISPR-based gene editing (Carballar-Lejarazú and James, 2017) (Dong et al., 2018).

Any refractory gene aiming to interrupt the development of the malaria parasite places a strong selective pressure on parasites capable of circumventing this interruption, and resistance mechanisms can be imagined for any of the effector genes produced thus far. For an antimalarial gene that prevents the parasite from recognizing the mosquito midgut by saturating the midgut receptors in the mosquito (Ito et al., 2002), parasites could evolve to recognize other receptors on the midgut, enabling them to pass into the hemocoel. Similarly, for a refractory gene that saturates parasite recognition receptors in the salivary glands (Ito et al., 2002), parasites could evolve to recognize other receptors, enabling them to pass through and to be injected into susceptible humans upon the mosquito taking a blood meal.

Melanization-based mechanisms that inactivate malaria parasites exist in nature, as do resistant mechanisms in the parasite, providing insight into parasite evolutionary responses. In nature, there is a melanization pathway by which pattern recognition receptors (PRRs) bind to pathogen-associated molecular patterns (PAMPs), activating a cascade of reactions culminating in large-scale melanin synthesis and encapsulation of the parasite in a melanin coat (Cerenius et al., 2008). Parasite resistance responses include: i) breaking down melanin, or ii) interfering with melanin activation or synthesis pathways. Yassine et al. have documented a mechanism whereby two key regulators of malaria melanization in *An. gambiae* (TEP1 and CLIPA8) are deactivated (Yassine et al., 2012). Mutations could occur on the PAMPs, preventing the PRRs from binding to them.

Care must be taken in the choice of disease-refractory genes to avoid a situation in which the effector gene selects for parasites displaying higher infectivity/pathogenicity in the population. Work on effector molecules based on single-chain antibodies suggests that these could prevent sporozoites from entering the salivary glands at infection levels encountered in natural conditions (Isaacs et al., 2012). However, in the laboratory, higher-than-normal parasitemia resulted in some parasites reaching the salivary glands despite the effector gene being present. Such effector genes could, in principle, select for parasites strains that reproduce at higher rates and produce higher loads, with potentially detrimental consequences for human disease burden and transmission. Clearly, in the presence of anti-parasite effector genes, malaria parasites displaying resistant phenotypes will be selected for in the population. The relevant question is therefore how we can substantially delay the emergence and subsequent spread of these parasite varieties. Perhaps the best approach might be to attack the parasites at as many stages as possible with an array of effector mechanisms to prevent the evolution of resistance, or increased infectivity/pathogenicity. That said, these effectors must be challenged with a diversity of parasites, similar to what is found in the wild, as opposed to a singular lab strain that has been maintained for many years in the lab (Isaacs et al., 2012). This may be the best lab-based attempt at generating data that could be translatable to what could occur in nature, however even this may still not reflect what would happen in the wild.

### Lessons Learned From Antiviral Drug Development and Vector-Virus Interaction Studies

Besides malaria, the largest vector-borne disease burden is that due to the family of arboviruses transmitted by *Ae. aegypti*, including DENV and ZIKV. *Ae. aegypti*-transmitted arboviruses are of particular concern at present, as the mosquito vector has rapidly expanded its range in recent years (Huang et al., 2017; Lee et al., 2019; Ryan et al., 2019). In response, a variety of approaches have recently been taken to engineer mosquitoes refractory to these diseases, including: i) transgene-based RNA interference (RNAi) in the midgut (Franz et al., 2006) and salivary glands (Mathur et al., 2010), ii) expression of synthetic miRNAs (Buchman et al., 2019b), iii) use of broadly neutralizing, single-chain variable fragments (scFv) (Buchman et al., 2019a), iv) use of antiviral hammerhead enzymes (Mishra et al., 2016), and v) transgenic activation of antiviral pathways (Jupatanakul et al., 2017). Nonetheless, these arboviruses are prone to rapidly evolving resistance to antiviral strategies. Lessons may be learned from human antiviral drug development and vector-virus immune studies, highlighting potential pitfalls that may transpire during antiviral effector development.

Most mosquito-borne viruses of human health concern are RNA viruses in the families *Flaviviridae*, *Togaviridae*, and *Bunyaviridae*. The high genetic variability and mutability of RNA viruses, due to low fidelity RNA-dependent RNA polymerase activity (Steinhauer et al., 1992), along with their high yield and fast replication times, drive the diverse host and environmental ranges of these viruses. This leads to a dynamic population of mutant viral genomes, referred to as a quasispecies, that can rapidly increase or decrease in population frequency according to their fitness (Domingo and Holland, 1997; Lauring and Andino, 2010; Andino and Domingo, 2015). At any point in time, the viral population could comprise one or more variants that have a resistant mutant to an otherwise strong selection pressure, such as a drug or antiviral effector.

These attributes of RNA viruses have confounded the development of antiviral drugs for many important human viral infections. For example, due to the high mutation frequency of the human immunodeficiency virus (HIV), a multi-target combinational drug approach is required to minimize HIV replication. This approach typically targets the viral reverse transcriptase (RT) gene, or other essential viral genes with reasonably high genetic or structural conservation (Ceccherini-Silberstein et al., 2005). However, resistance to these drugs is still frequent and often mechanistically distinct (Menéndez-Arias, 2013). Although not strictly comparable, this example demonstrates that targeting multiple essential viral genes and conserved sites in the viral genome, the common approach taken to mitigate antiviral effector resistance in mosquitoes, does not necessarily prevent resistance from emerging.

A multitude of clinical and laboratory-based examples exist of similar RNA viruses evolving drug resistance despite mitigative design considerations. For instance, a multi-target approach did not prevent rapid viral resistance to ribavirin (Pfeiffer and Kirkegaard, 2003; Vignuzzi et al., 2005; Beaucourt and Vignuzzi, 2014; Debing et al., 2014), an antiviral drug that targets and mutates RNA viruses at multiple sites and time points during the viral replication cycle (Crotty et al., 2001; Crotty et al., 2002). Furthermore, when target site mutations have a high fitness cost, RNA viruses such as influenza have been shown to develop compensatory mutations to improve their fitness (Bloom et al., 2010; Bloom et al., 2011; Behera et al., 2015). Compensatory mutations have also been identified in other RNA viruses, notably increasing replication efficiency in large-deletion, replication-deficient CHIKV strains in both primate and mosquito cell cultures (Nougairede et al., 2013). However, these problems are not insurmountable, as the drugs described in these examples are still effective in combined and selectively administered antiviral therapies.

Evolution of resistance to antiviral effectors is to be expected in the event that population replacement technologies are implemented to combat *Ae. aegypti*-transmitted arboviruses. Mosquitoes have evolved numerous systemic, small RNA-mediated immune defense mechanisms against viral infections that drive adaptation of viral counter-defenses. Notably, viral-associated immune suppression has been identified in laboratory studies of CHIKV infected mosquitoes and mosquito cell lines (McFarlane et al., 2014; Mathur et al., 2016) and in ZIKV vertebrate host models (Xia et al., 2018; Zheng et al., 2018). Presumably, similar viral counter-defenses could be developed against antiviral effectors. In prominent disease vectors, such as *Ae. aegypti*, the immune response is not adequate to prevent the replication and dissemination of arboviruses, and consequently allows systemic infections that lead to arboviral transmission to vertebrate hosts. One future approach to antiviral effector design could be to enhance natural mosquito immunity. Another approach could be to circumvent viral evasion mechanisms in order to support viral clearance (Li and Ding, 2006).

The rapid mutation rates of arboviruses are key to their broad host tropism. Host-specific selection pressure can constrain viral diversity (Weaver et al., 1992; Coffey et al., 2008; Vasilakis et al., 2009; Grubaugh et al., 2016), and could be advantageous in effector design. Effectors that act very early will have the advantage of targeting smaller, less diverse viral populations, and hence viral resistance will be slower to emerge. However, these viral populations rapidly diversify in the vector given their high mutation rates and large population sizes. Virus host adaptations are found frequently in nature (Brault et al., 2004; Moudy et al., 2007; Tsetsarkin and Weaver, 2011) and can initiate or amplify disease outbreaks (Feng et al., 2019), as seen in the 2005-2006 CHIKV outbreak in La Réunion (Tsetsarkin et al., 2007). As the virus disseminates in the mosquito, viral diversity may be further bottlenecked (Patterson et al., 2018) and therefore additional tissue and timing-appropriate effector strategies may be able to take advantage of the lower viral diversity at these later bottlenecks in the transmission cycle.

These examples indicate the immense potential to rapidly constrain the host range of these viruses, and effectors that disrupt areas of the viral genome required for host switching could further limit their epidemic potential. Many conserved, host-essential regions have been discovered in arboviruses, e.g., the 3’UTR of DENV (Villordo et al., 2015; Filomatori et al., 2017) and other arboviruses (Hyde et al., 2015; Morley et al., 2018). While host-specific selection pressures restrict the evolution of arboviruses in the mosquito, the immunity and host-associated diversification of arboviruses in mosquitoes indicate strong potential to develop resistance to antiviral effectors. Studying these important vector-virus interactions and their role in viral evolution will support the design of new and more robust antiviral effectors. Furthermore, with continued advancements in genome engineering, effector design capabilities will also improve. In the next section, we discuss the current state of antiviral effector development and future directions for these effectors.

### Arbovirus-Refractory Genes and Resistance Concerns

The majority of arbovirus-refractory effectors developed to date utilize RNAi-based approaches (Sánchez-Vargas et al., 2009; McFarlane et al., 2014; Blair and Olson, 2015). These have been used to target DENV serotype 2 (DENV-2) for over a decade, with trailblazing studies demonstrating that transgenes encoding long double-stranded RNAs serve as small interfering RNAs (siRNAs) capable of targeting the viral genome and consequently suppressing viral replication and transmission (Franz et al., 2006; Travanty et al., 2004; Mathur et al., 2010; Franz et al., 2014). Similar approaches have recently been implemented against DENV serotype 3 (DENV-3), CHIKV and ZIKV (Yen et al., 2018; Buchman et al., 2019b). Yen et al. (2018) developed transgenes encoding microRNAs (miRNAs)—a class of small noncoding RNAs involved in the regulation of gene expression—targeting DENV-3 and CHIKV, triggered either ubiquitously or in the midgut in response to a blood meal. These effectors still need to be optimized; however, reductions in DENV-3 and CHIKV transmission rates were significant—∼94 and ∼77–83%, respectively. Buchman et al. (2019b) developed transgenes encoding a cluster of synthetic small RNAs designed to target the ZIKV genome in the midgut (Buchman et al., 2019b). Results for these effectors were impressive, with homozygotes being completely refractory to ZIKV infection, and heterozygotes having infection and transmission rates low enough to make them unlikely to transmit to a susceptible human host.

All of these approaches are based on small RNAs, and to prevent the evolution of viral escape variants, high target coverage of the viral genome is required. Indeed, some viruses have been shown to replicate to high enough titers that they can overcome an RNAi response (McFarlane et al., 2014), and engineering efforts must ensure that high viral replication is not an evolutionary consequence. The rigorous target sequence conservation required in parts of the small RNA-targeting sequence, and the high mutation rate of RNA viruses, have resulted in the rapid development of resistance to other RNAi technologies (Boden et al., 2003; Das et al., 2004; Gitlin et al., 2005; Westerhout et al., 2005; Wilson and Richardson, 2005; Nishitsuji et al., 2006; Wu et al., 2011). The solution of Buchman et al. (2019b) was to use eight separate small RNAs to target 6–10 conserved protein-coding genes in the ZIKV genome, reducing the possibility of escape mutants.

An RNAi-mediated effector itself may also increase viral genetic diversity thereby potentiating viral escape. For example, the RNAi immune response drove the diversification of West Nile virus in mosquitoes (Brackney et al., 2009) and mosquito cells (Brackney et al., 2015). It is unknown whether effectors that use a similar RNAi-mediated antiviral mechanism will drive diversification of arboviruses, and if so, what the impact of this diversification would be on viral resistance. It is also still an open question as to how many small RNAs are necessary to prevent viral escape mutants in a diverse wild population, and the potential effect of this RNAi-mediated approach on viral evolution.

A recent approach taken to target multiple serotypes of DENV (1-4) utilized a broadly neutralizing scFV (Buchman et al., 2019a), reducing viral infection and dissemination of all four major serotypes of DENV. Another approach to minimize the emergence of escape mutants is to use small antiviral hammerhead ribozymes to increase the number of sites being targeted in the viral genome. This was used by Mishra et al. (2016) to target the CHIKV genome (Mishra et al., 2016). Error-prone activities of RNA polymerase provide opportunities for viruses to escape from ribozyme catalysis (Scherer and Rossi, 2003); however this can be overcome by using antiviral group-I introns, and by targeting conserved sequences in arboviruses (Carter et al., 2015). As discussed above, these designs must consider the high mutation rate and large size of viral populations, or viral “quasispecies”, and the implications of these features on viral resistance to effector genes. That said, off-target effects of small antiviral RNAs may help to reduce the risk of escape mutants, despite the heightened diversity of viral quasispecies (Yen et al., 2018).

Taken together, arboviruses and their corresponding effector genes clearly have distinct properties as compared to the malaria parasites and antimalarial effectors; however the critical related question remains as to how we can substantially delay the emergence and spread of viral escape mutants.

### Delaying and Managing Resistance to Disease-Refractory Genes

Strategies for delaying and managing resistance to disease-refractory effector genes in mosquitoes parallel those for antimalarial and antiviral drugs in humans (White and Olliaro, 1996). Three key elements include: i) preventing emergence of resistance, ii) monitoring efficacy and confirming resistance when present, and iii) managing resistance through containing its spread and ensuring a pipeline of new effector genes.

**Figure 2:**
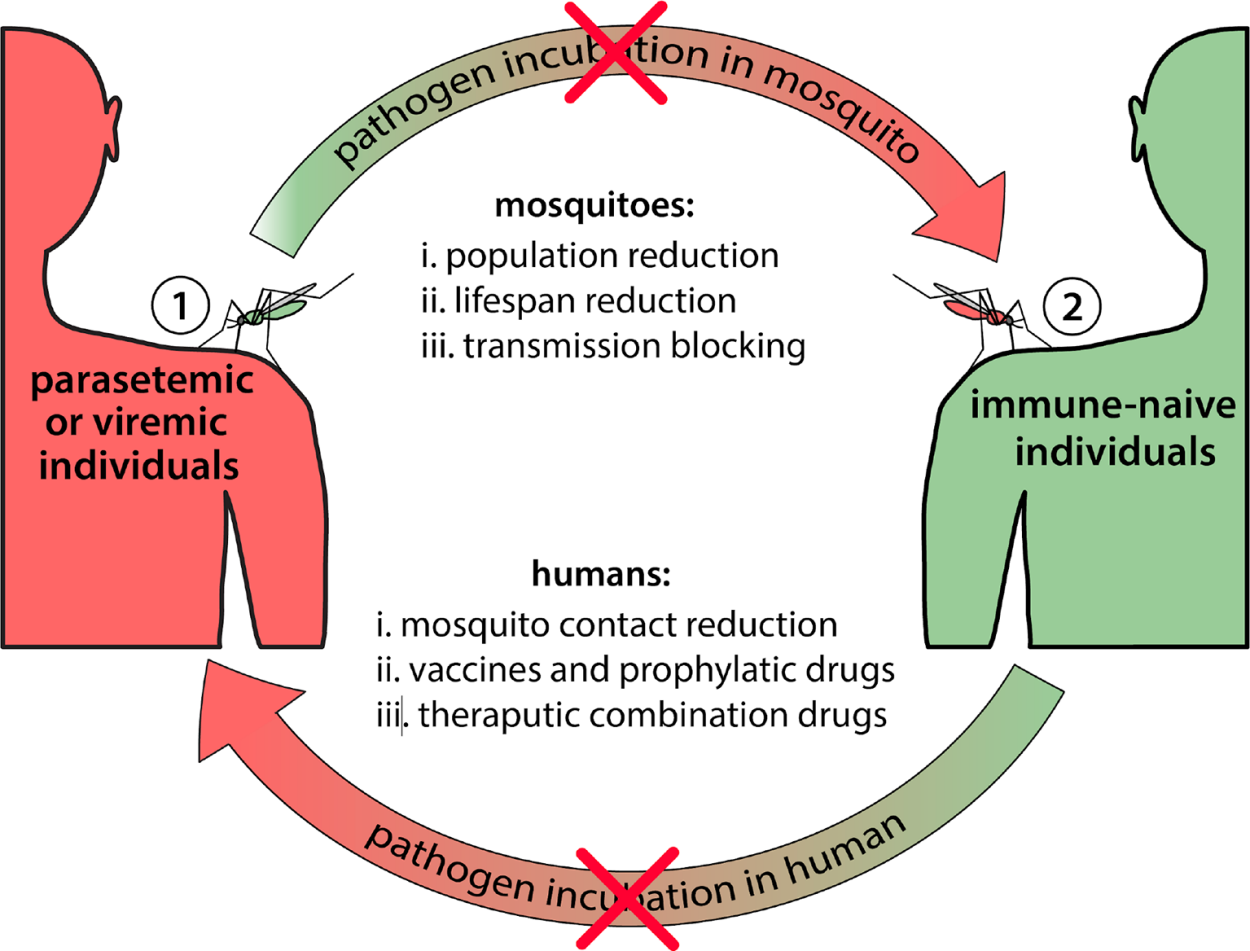
Potential intervention points to block mosquito-borne disease transmission. Mosquito-borne diseases require incubation periods in both mosquito (extrinsic) and human (intrinsic) hosts. Blocking the cycle at any point will interrupt transmission. Multiple coordinated interventions will likely be needed to achieve effective and sustainable disease control. Potential interventions that could be implemented on the mosquito vector include: i) mosquito population reduction strategies (e.g. insecticide application, and the sterile insect technique), ii) mosquito lifespan reduction strategies (e.g. *Wolbachia*-associated lifespan reduction), and iii) pathogen transmission blocking in mosquitoes (e.g. population replacement gene drive systems, or population transfection with *Wolbachia*). Potential interventions that could be implemented on the human host include: i) human-mosquito contact reduction (e.g. insecticide-treated bed nets, and spatial repellents), and ii) pathogen transmission-blocking in humans using vaccines and prophylactic drugs, and iii) combination-therapy drugs. Reducing pathogen transmission, and hence the pathogen population size, reduces the ability of the pathogen to evolve resistance to effector genes.

**Figure 3:**
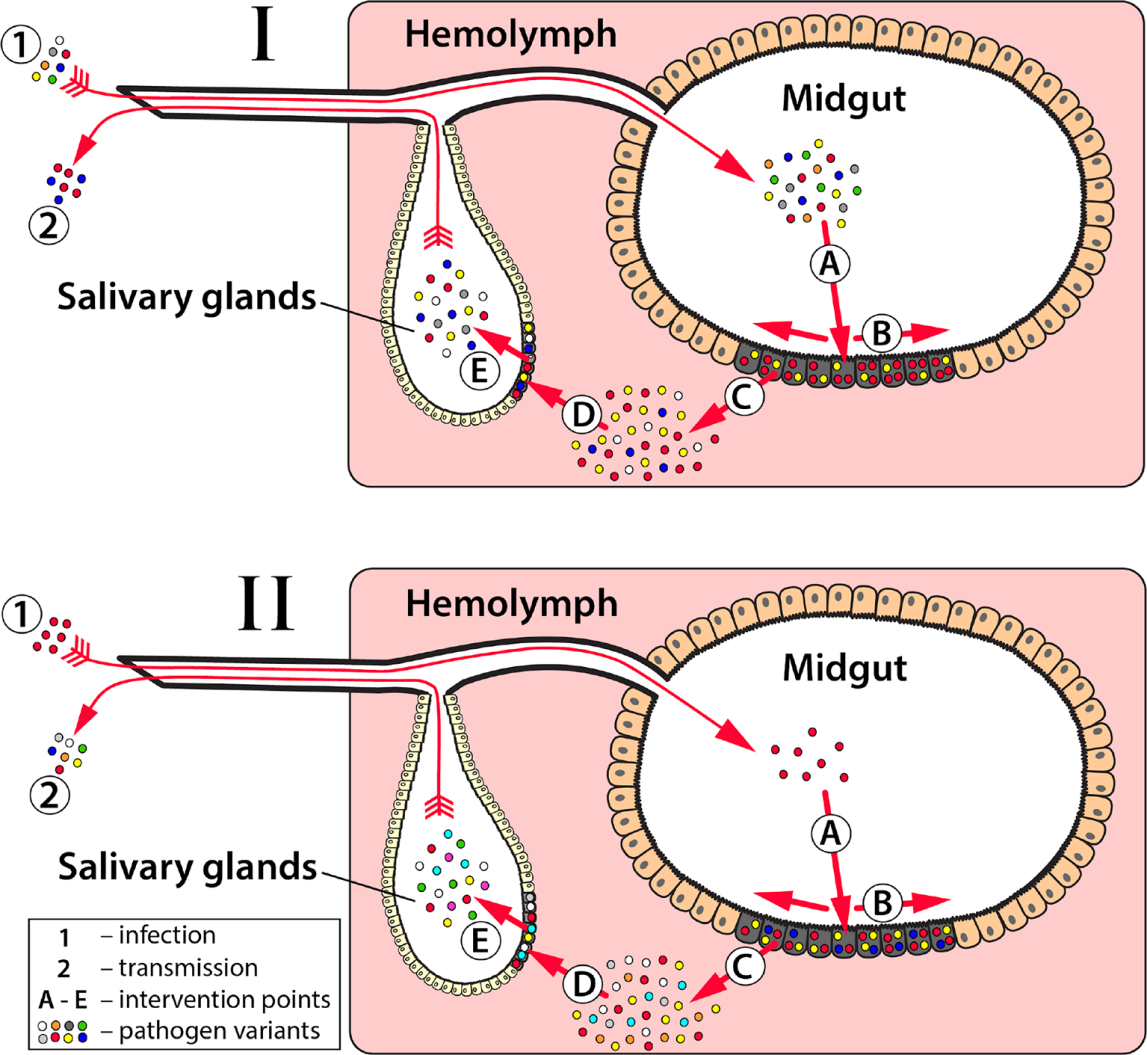
Key events in pathogen transmission from mosquito to vertebrate host and implications to effector selection. The transmission of mosquito-borne pathogens can be suppressed by blocking one or more of the steps required for pathogen infection and/or dissemination in mosquitoes. For *Plasmodium* parasites (I) and arboviruses (II) effectors can be built to block one or more essential points in the pathogen replication cycle: **(A)**—initial infection of a midgut epithelial cell; **(B)**—spread of infection among midgut epithelial cells; **(C)**—midgut escape of infecting agents from midgut epithelial cells into hemolymph; **(D)**—salivary gland infection, and **(E)**—salivary gland escape where the pathogens spread to salivary glands and are expectorated in a subsequent blood meal of the female mosquito, respectively. If any one or more of these events is completely blocked, then transmission cannot occur. At each of these potential intervention points, there is a bottleneck that constricts pathogen population size and diversity. By selecting effectors with timing and specificity that can exploit these natural bottlenecks, it may slow the rate that pathogen resistance to the effector is developed. For example, as *Plasmodium* parasites (I) move through the gametocyte, gamete, zygote **(A)**, ookinete **(B)**, and oocyst **(B-C)** stages, their population size and diversity progressively decreases with a substantial genetic bottleneck at each stage. However, the population size increases dramatically when the oocysts release sporozoites **(C-E)**. Most current anti-*Plasmodium* effectors developed to date target the ookinete stage when the parasite population is relatively small, but if pathogen resistance to anti-ookinete effectors does occur then targeting the oocyst stage, where the parasite population size is the lowest and least diverse, may slow resistance. Similar to a combinatorial drug approach, targeting multiple parasite development stages concurrently is also a strategy that researchers are taking to circumvent resistance. Arbovirus populations (II) go through a series of population expansions and reductions that correspond with the essential points in their replication cycle **(A-E)**. In the vertebrate, purifying selection reduces the genetic diversity of viruses acquired during blood feeding (1), but with the high mutation rate the genetic composition of the virus genome drifts as it disseminations through the mosquito. Effectors that target the virus in the early stages of infection **(A** and early **B)** prior to expansion and diversification of the virus in the midgut would potentially decrease the likelihood of the virus circumventing an antiviral effector. Most current antiviral effector designs target these early stages.

Prevention or slowing of the emergence of effector resistance is the first step to ensure effectors are effective components of population replacement strategies. This has two dimensions: i) increasing the evolutionary hurdle required by the pathogen to evolve resistance to the effector gene, and ii) reducing the size of the pathogen population to reduce the rate of resistance emergence (**Figure 2**). Most of the discussion thus far has focused on the former. Strategies to increase the evolutionary hurdle include: i) using multiple effectors that attack the pathogen at multiple sites on its genome and/or multiple stages of development (**Figure 3**) (Mishra et al., 2016; Buchman et al., 2019b), and ii) ensuring that the effectors used are effective at substantially reducing pathogen transmission, and in a wide range of individuals representing the genetic diversity of the species. As mentioned earlier, a crucial goal is to prevent selection of escape mutants with a higher viral or parasitic load. This must be true across the species range of the vector, as ambitious population replacement strategies aim to introduce disease-refractory genes on a potentially continental scale, and hence the effector genes must be robust to pathogen evolution on a similar scale.

Reducing the size of the pathogen population by other means is complementary to the design of robust effector genes, and provides an additional rationale for maintaining an integrated vector and disease control program. The simple rationale is that, if the pathogen population is smaller, then there will be less opportunities for effector resistance to emerge. For malaria, additional approaches to reduce the parasite burden at the population level include: i) vector control methods, such as the distribution and use of insecticide-treated nets and spraying of walls with insecticides, and ii) the distribution of antimalarial drugs (ACTs), either when cases present at health facilities, or through some variety of mass drug administration. A range of novel vector control interventions are also becoming available to further reduce vector densities (Killeen et al., 2017; Kiware et al., 2017). For arboviruses, additional approaches rely on vector control, traditionally through fogging with insecticides, or larval source reduction, but more recently through the use of *Wolbachia*-infected mosquitoes, either as a form of population suppression or replacement (Hoffmann et al., 2011), and the release of genetically sterile males as a form of population suppression (Carvalho et al., 2015). Models of disease transmission between vectors and humans, as well as vector population dynamics(Killeen et al., 2017; Kiware et al., 2017) and pathogen strain evolution, will allow the relative contributions of these complementary strategies to be assessed in future modeling studies. Data to inform these models is limited at present; however initial impressions may be gained by including sensitivity analyses, and will improve as further data is obtained, for instance, pertaining to pathogen population size and genetic diversity.

Upon implementation, routine monitoring will be integral to preventing the spread of pathogen resistance to effector genes. Early detection through regular monitoring allows alternative effectors to be introduced into the vector population and/or alternative vector and disease control interventions to be put in place to minimize the selection and further spread of effector resistance. To aid in these efforts, accurate rapid assays for pathogen susceptibility to effector genes will be extremely useful, as will a pipeline of alternative anti-pathogen effector genes, and a resistance management strategy including geographical considerations informed by spatial modeling.

Clearly, considerations regarding the evolutionary robustness of anti-pathogen effector genes are the most urgent, and progress made here will ease the load of resistance management later; however, as this technology moves closer to the field, the emphasis will quickly shift to monitoring and management.

### Target Product Profile Considerations for Duration of Efficacy

If the competition between effector gene design and pathogen evolution is a tug-of-war, then the TPP would determine the position of the line that one has to pull the other over. Where we draw that line is determined by what our goals and evaluation criteria are. Are they entomological or epidemiological? If entomological, what is the minimum proportion of the vector population we require to be disease-refractory, and for how long? If epidemiological, what is the desired reduction in clinical incidence of disease (or some other disease-related measure), and over what time period? And what vector species and pathogens are we focusing our attention on?

Likely, there will be multiple TPPs for different stages of technology implementation. Leading up to the first phase of field trials, an entomological endpoint may be more feasible, and it may only be necessary to have a product capable of being prevalent and functional in a local vector population for a few years, i.e., over the duration and spatial extent of a field trial (James et al., 2018). Leading up to an open release with the intent of wide-scale disease control, however, an epidemiological endpoint will be of most interest, and it will be necessary to have a product capable of spreading widely and retaining its effectiveness for a duration potentially required to achieve widespread pathogen elimination (James et al., 2018) and measured at timepoints sufficiently after the release to see an effect on disease incidence.

Which TPP we are aiming for dramatically influences several minimal properties of the drive system including: i) for homing-based drive systems, the rates of accurate homology-directed repair and generation of homing-resistant alleles, ii) the rates of mutational loss of the gene drive system and/or effector genes, and iii) the rate of pathogen evolution in response to the effector genes, among other properties. Here, we limit our attention to the latter property regarding pathogen evolution; however, all are essential to achieve the desired entomological and/or epidemiological endpoints. To be effective in achieving wide-scale disease control and potentially elimination through reduced pathogen transmission in a range of vectors representing the genetic diversity of the species, multiple effectors that attack the pathogen at multiple genomic sites and/or stages of development will need to be included (Mishra et al., 2016; Buchman et al., 2019b). It will also be essential to reduce the parasite population by other means, including vector control and, for malaria, the distribution of antimalarial drugs (Feachem et al., 2019).

## Conclusion

The advent of CRISPR-based gene editing has greatly expanded the realm of possibility for engineering of disease-refractory effector genes. Combined with monitoring and rapid management of pathogen resistance when it emerges, we believe that the odds are increasingly favoring the effector genes over the pathogens in this forthcoming evolutionary tug-of-war. In the coming years, we expect more anti-pathogen effectors to be developed, thereby increasing the array of tools available to combat these pathogens. Concomitantly, laboratory and modeling studies of how to best prevent the emergence of resistance in the field should be conducted to investigate ways to create “evolution-proof” strategies for the development and release of effector gene products. Semi-field studies and mathematical modeling will both have a role in highlighting challenges and opportunities for these systems, as they have for drug and insecticide resistance (Koella et al., 2009; Wale et al., 2017). As these technologies are developed and improved and regulatory approvals are granted, we remain optimistic they will play a pivotal role in eliminating vector-borne disease transmission on a global scale, in concert with other strategies (Feachem et al., 2019).

## Author Contributions

All authors contributed to the writing of this review.

## Funding

This work was supported by funding from the Defense Advanced Research Project Agency (DARPA) Safe Genes Program Grant (HR0011-17-2- 0047) awarded to O.S.A. and J.M.M. Funds from the UC Irvine Malaria Initiative supported J.M.M.

## Conflict of Interest

The authors declare that the research was conducted in the absence of any commercial or financial relationships that could be construed as a potential conflict of interest.
